# Book Reviews

**Published:** 1872-04-01

**Authors:** 


### Book Reviews.

Plain Talk about Insanity; its causes, forms, symptoms, and the treatment of mental diseases. With remarks on hospitals and asylums, and the medico-legal aspect of insanity. By T. W. Fisher, M.D. Boston: Alexander Moore. 1872.

This is a monograph of nearly 100 pages, written as much for the public as the profession. It contains much useful information and many valuable suggestions regarding the nature and management of insanity.

For sale by W. B. Keen, Cooke & Co., Chicago. Price, $1.50.

Animal and Vegetable Parasites of the Human Skin and Hair. By B. Joy Jeffries, A.M., M.D., Fellow of the Massachusetts Medical Society, etc., etc., etc. Boston: Alexander Moore. 1872.

This is a neatly published duodecimo of 102 pages. The author appears thoroughly conversant with the subject, and has furnished us with a very interesting and valuable elementary treatise on the animal and vegetable parasites that infest the human hair and skin.

For sale by W. B. Keen, Cooke & Co.

Anæsthesia, Hospitalism, Hermaphroditism, and Proposal to stamp out Small-Pox and other Contagious Diseases. By Sir James Y. Simpson, Bart., M.D., D.C.L., late professor of Midwifery in the University of Edinburgh. Edited by Sir W. G. Simpson, Bart., B.A. New York: D. Appleton and Co., 549 Broadway. 1872.

This is a volume of 562 pages published in good style. It contains essentially all that the late Sir James Y. Simpson wrote at various times concerning the topics in the title page. It is a collection and arrangement in one volume of numerous essays and papers, contributed from time to time by the gifted author, and published originally in various periodicals. The first 288 pages are occupied with the subject of Anæsthesia; embracing its History, Defenses, the Nature and Power of various Anæsthetics; Applications of Anæsthesia in Surgery and Medicine; in Midwifery; and Local Anæsthesia.

The subject of Hospitalism occupies 118 pages. This part of the volume contains some valuable statistics in reference to the results of surgical operations in large metropolitan hospitals and in rural districts, and much that is valuable in relation to the causes of mortality in hospitals, and in relation to improved methods for constructing such institutions. Thirty-three pages are devoted to the subject of Hermaphroditism.

The last ten pages are devoted to the subject of Preventing Small-Pox and other Contagious diseases. The chief means advocated are isolation, disinfection, and cleanliness. The book will be found one of the most convenient and valuable that the physician can have in his library. The editor, though not a physician, has certainly conferred a great favor on the profession by collecting and publishing the valuable writings of his father in one convenient volume.

Transactions of the Medical Society of the State of New York for the year 1870. Albany. New York.

This is a bound volume of over 400 pages, containing the transactions and papers of the New York State Medical Society at the annual meeting of February, 1870. There are a number of valuable papers, and much information in regard to the topography and diseases of various localities in that State, and a most excellent steel plate likeness of the late Alden March, M.D., etc., of Albany, N. Y. We have not time or space to analyze any of the papers at present.

Dr. Boise recommends liquid carbolic acid as an application to condylomater.

